# A Rare Presentation of Multiple Myeloma With Spontaneous Haematoma

**DOI:** 10.7759/cureus.64077

**Published:** 2024-07-08

**Authors:** Adeel A Jafri, Swarnima K. C., Elena Ioannou, Johnson Philippananthan

**Affiliations:** 1 Medical Oncology, Sheffield Teaching Hospitals NHS Foundation Trust, Sheffield, GBR; 2 General Medicine, University Hospital Lewisham, London, GBR; 3 General Medicine, Luton and Dunstable University Hospital, Luton, GBR; 4 Geriatrics, Luton and Dunstable University Hospital, Luton, GBR

**Keywords:** hyperviscosity syndrome, diagnosis of rare cases, immunoglobulin g, haematoma, multiple myeloma

## Abstract

Multiple myeloma (MM) typically presents with characteristic symptoms such as bone pain, hypercalcaemia, renal dysfunction, and anaemia. However, atypical presentations of MM, though rare, have been reported. These atypical presentations pose a diagnostic challenge due to their varied clinical manifestations, leading to potential delays in diagnosis and treatment initiation.

We present the case of a 75-year-old woman who presented to the emergency department with a spontaneous haematoma on the dorsal aspect of her left hand and wrist. Despite lacking classical symptoms of MM, such as bone pain or renal dysfunction, laboratory investigations revealed abnormal findings, including high serum protein levels, low albumin, and abnormal immunoglobulin levels. Serum protein electrophoresis and immunotyping confirmed a diagnosis of MM, specifically the immunoglobulin-G lambda type. Additionally, urine protein electrophoresis further supported the diagnosis. Imaging studies did not show radiological evidence of myeloma.

The absence of classical symptoms in our patient underscores the importance of considering MM in the differential diagnosis of atypical cutaneous presentations. Laboratory investigations, particularly serum protein electrophoresis and immunotyping, played a crucial role in establishing the diagnosis. The patient was treated with pulsed dexamethasone and plasmapheresis, followed by initiation of VCD chemotherapy protocol.

Atypical presentations of MM present diagnostic challenges for clinicians. Our case highlights the importance of maintaining a high index of suspicion for MM, even in the absence of classical symptoms. Early recognition and diagnosis are essential for prompt management and improved patient outcomes. Clinicians should remain vigilant for atypical presentations of MM to ensure timely intervention and treatment initiation.

## Introduction

Multiple myeloma (MM), while a rare haematological neoplastic condition, has shown an increase in age-standardised incidence rates for females and males combined by 33% in the UK between 1993-1995 and 2016-2018 [1]. It is characterised by plasma cell proliferation in the bone marrow producing monoclonal immunoglobulins. This typically causes extensive skeletal destruction with osteolytic lesions, osteopenia, pathological fractures, hypercalcaemia, unexplained anaemia and renal failure. Atypical presentations such as cutaneous symptoms, ocular involvement, and gastrointestinal manifestations are also documented, though they occur less frequently. In patients with newly diagnosed MM, the reported incidence of hyperviscosity syndrome (HVS) ranges from 0.5% to 4.8%. Among these cases, cutaneous manifestations are observed in approximately 24% [2]. Such statistics underscore the necessity for clinicians to consider a broad differential diagnosis when evaluating patients with unexplained haematological or systemic symptoms.

Hyperviscosity is an increase in the internal resistance of blood in the body, resulting in a reduction of its flow. It accounts for only 2% of MM, which is usually associated with immunoglobulin M (IgM) [3]. HVS can manifest with bleeding symptoms due to impaired platelet aggregation, neurological symptoms and visual disturbance. One way this occurs is by a deformity of the shape of red blood cells (such as in sickle cell disease and spherocytosis). Another is by any pathological elevation of the components of blood, either cellular (red blood cells, white blood cells, platelets) or acellular (serum proteins) [4].

Hypergammaglobulinemia is the most common cause of HVS, especially the monoclonal condition of Waldenström macroglobulinemia (WM), as the protein is a pentamer and so larger. This is followed by myelomas, with the immunoglobulin-G (IgG) type accounting for less than 5% of the cases [4]. One of the significant effects of hyperviscosity is its impact on platelet function. The high concentration of immunoglobulins can interfere with platelet aggregation by coating the platelet surface and hindering their ability to stick together. This leads to a failure of platelet aggregation, which is crucial for the formation of a haemostatic plug in response to vascular injury. Consequently, patients with HVS may present with bleeding tendencies, such as mucosal bleeding or spontaneous haematomas, as seen in our patient.

## Case presentation

A 75-year-old woman presented to the emergency department with a spontaneous haematoma associated with swelling over the dorsal aspect of the left hand and wrist. Her past medical history included a previous tissue aortic valve replacement (June 2021), not on anti-coagulation, and carcinoma of the left breast (2018) for which she had undergone a left wide local excision with sentinel node biopsy, followed by adjuvant radiotherapy and anastrozole treatment.

Physical examination

**Figure 1:**
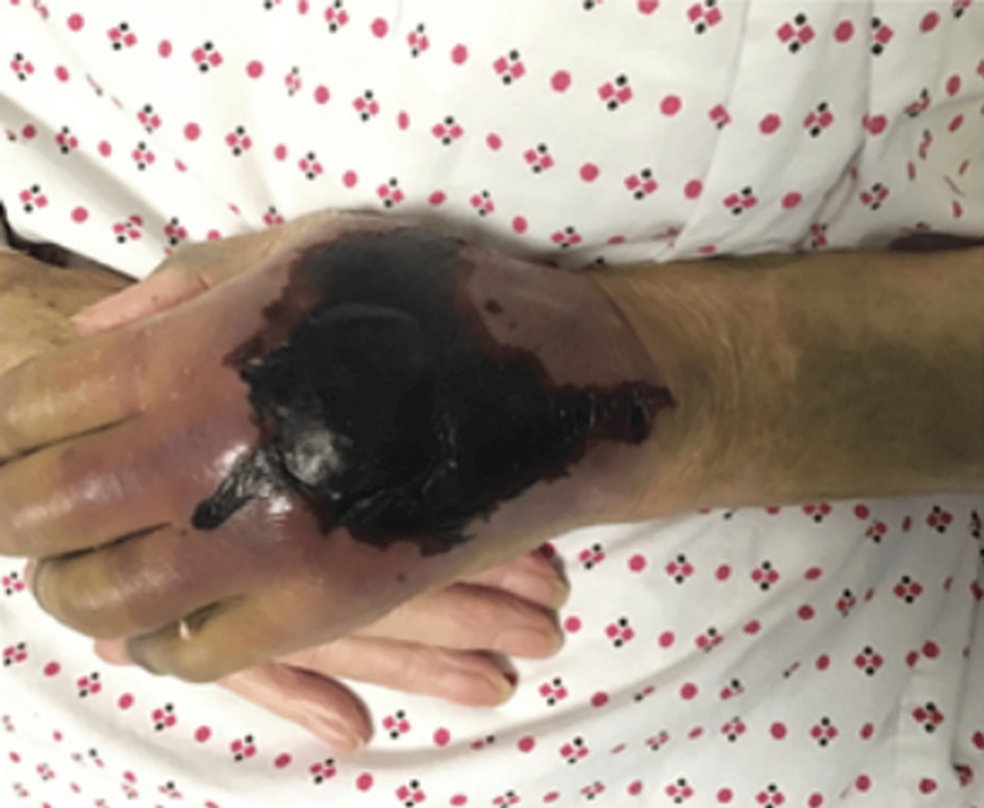
Subcutaneous haematoma over the left hand

There was swelling and bruising affecting the left wrist and dorsum of the hand (Figure 1), with normal capillary refill, good peripheral pulses and normal joint movements. The patient's blood pressure was measured at 177/88 mmHg. Bi-basal crepitations and a systolic murmur were heard on auscultation. Cranial nerves were intact, and neurological examinations were normal.

Laboratory examinations

A high total protein count along with low albumin was noted. Haemoglobin and platelets were also below lower limits. Liver function tests and electrolytes were within the normal range (Table 1).

**Table 1:** Initial routine bloods panel * indicates abnormal blood investigations. APTT: activated partial thromboplastin time; INR: international normalised ratio; LDH: lactate dehydrogenase

Investigations	Results	Reference Ranges
Haemoglobin (g/L)	86*	129-160
Platelets (x10^9 ^L)	103*	150-450
White Cell Count (x10^9 ^L)	5.5	4-11
Prothrombin Time (secs)	17.1	9-14
APTT (secs)	37	26-38
INR	1.4	0.8-1.2
Adj. Calcium (mmol/L)	2.4	2.2-2.6
Albumin (g/L)	20*	35-50
Total Protein (g/L)	157*	60-80
Creatinine (umol/L)	94	44-80
LDH (U/L)	357	<250

Immunology testing

Serum protein electrophoresis (SPE) and immunotyping demonstrated a serum paraprotein concentration of 94 g/L (66% serum paraprotein concentration). On immunofixation, the paraprotein band was identified as IgG lambda, with serum-free lambda chains measuring 320 mg/L (reference range 5.7-26.3 mg/L). Further immunoglobulin analysis showed immunoglobulin A 0.06 g/L (reference range 0.7-4.0 g/L), immunoglobulin M 0.04 g/L (reference range 0.4-2.3 g/L) and beta-2 microglobulin 3.6 mg/L (reference range <3 mg/L).

Peripheral blood film examination

**Figure 2:**
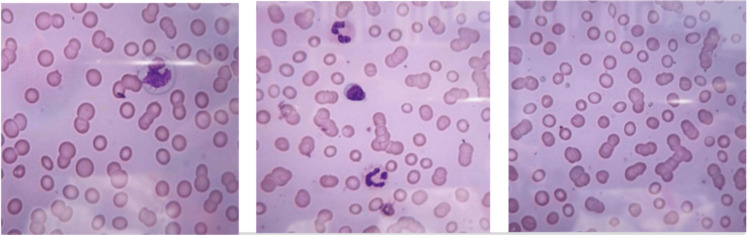
Blood film showing red cell anisocytosis (left), Rouleaux formation (centre) and reduced platelets (right).

The blood film reported red cell anisocytosis, Rouleaux formation and reduced platelets (Figure 2).

Urine investigations revealed a total protein of 0.17 g/L (reference range <0.15) and urine protein electrophoresis showed the presence of a band in the gamma region which was confirmed and identified as IgG lambda on immunofixation. All her serology tests including urinary legionella and pneumococcus screening were negative.

Imaging examinations

Additional investigations included a CT skeletal survey which showed no radiological evidence of myeloma and a dental screening with an orthopantomogram X-ray which ruled out osteonecrosis prior to starting bisphosphonate treatment.

## Discussion

In this case, the patient's initial presentation with a spontaneous subcutaneous haematoma and the absence of classical myeloma-related symptoms posed a diagnostic challenge. Given the wide range of potential presentations, it is crucial for clinicians to maintain a high index of suspicion for MM in patients presenting with atypical symptoms. Key clinical signs that should prompt consideration of MM include (a) unexplained anaemia: persistent anaemia not attributable to other common causes should raise suspicion; (b) bone pain or fractures: especially if there is no clear history of trauma; (c) renal dysfunction: unexplained renal impairment or proteinuria; (d) hypercalcemia: elevated calcium levels without other obvious causes; (e) neurological symptoms: such as peripheral neuropathy or symptoms indicative of spinal cord compression; (f) recurrent infections: frequent infections due to immune system compromise.

Laboratory investigations, including SPE and immunotyping, also play a crucial role in establishing the diagnosis. The International Myeloma Working Group recommends a screening panel of SPE, immunofixation electrophoresis (IFE), and free light chain (FLC) assays that give a combined sensitivity of 97.4% for screening monoclonal gammopathies [5]. The presence of a high serum paraprotein concentration of 94 g/L, identified as IgG lambda, along with abnormal immunoglobulin levels and peripheral blood film findings, provided valuable diagnostic clues. Furthermore, urine protein electrophoresis confirmed the presence of IgG lambda, reinforcing the haematological nature of the disorder.

Although laboratory evidence of high serum viscosity establishes the diagnosis, not all laboratories perform this test. Therefore, a diagnosis of MM is usually made based on the clinical picture when a potential cause is present or highly suspected. Further testing should always include a complete blood count, full serum chemistries, coagulation profile, and urinalysis [6]. The clinical signs of coagulopathy and investigation findings supported the diagnosis of HVS as a presenting feature of new MM. Following discussion with haematology, the patient was commenced on pulsed dexamethasone 20 mg daily and was transferred to a tertiary care centre for plasmapheresis treatment.

**Figure 3:**
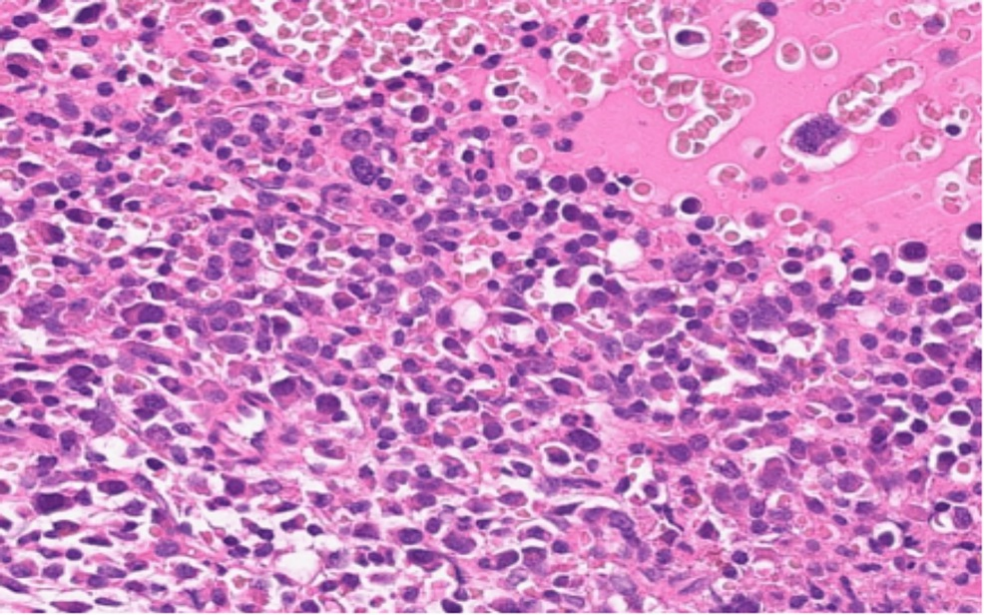
Bone marrow aspiration showing myeloma cells.

**Figure 4:**
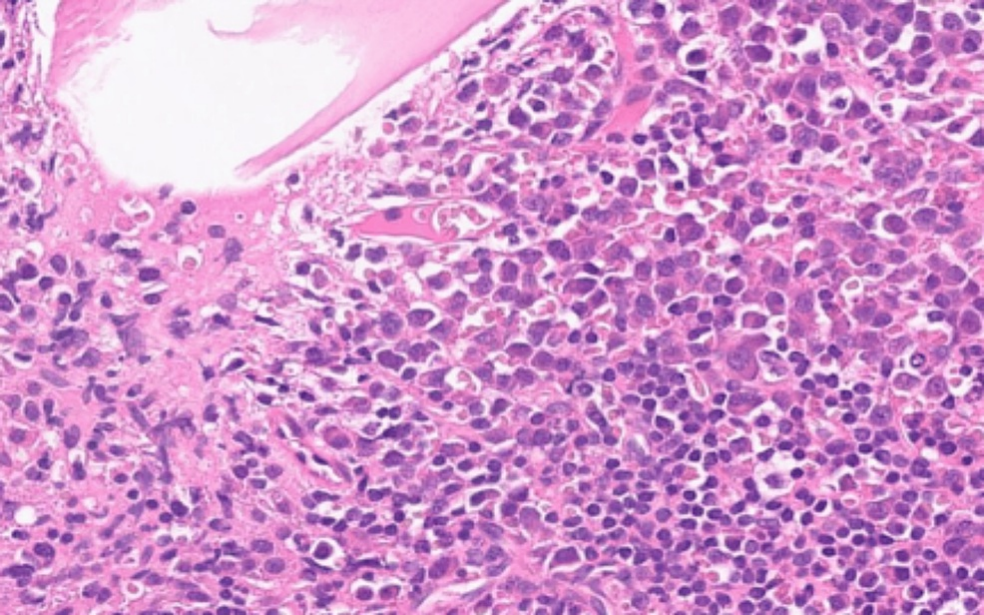
The figure showing typical advanced plasma cell infiltration.

Following plasmapheresis, there was a reduction in total protein levels from 157 g/L to 87 g/L and serum paraprotein levels from 94 g/L to 41 g/L. The patient was repatriated back to the district general hospital where a bone marrow trephine biopsy was performed. The neoplastic, pleomorphic plasma cells (Figure 3) were CD138 +ve, CD1 -ve, CD56 -ve and lambda light chain restricted. These results demonstrate an extensive clonal plasma cell infiltrate (Figure 4) in keeping with plasma cell myeloma, with tumour burden being 70% of total nucleated cells.

As guided by the haemato-oncological multidisciplinary team (MDT), the patient was started on VCD chemotherapy protocol (Velcade, Cyclophosphamide, Dexamethasone). Chemotherapy was discontinued after three cycles due to treatment-related side effects and the patient was then counselled for best supportive care. She passed away four months later at a local hospice due to worsening renal function leading to renal failure.

A case review by Tathineni et al. [1] in 2020 compiled a comprehensive list of uncommon presentations of MM. These included orbital presentations such as proptosis, gastrointestinal presentations such as acute pancreatitis and neurological presentations like cranial nerve palsies and unilateral hemiparesis. Despite this, we continue to find rarer presentations documented in this space such as an initial presentation with bilateral carpal tunnel syndrome [7].

## Conclusions

Atypical presentations of MM challenge every clinician to diagnose the condition. Raising awareness among clinicians about such rare cutaneous manifestations is essential for early diagnosis and prompt management. This case highlights a rare presentation of MM with HVS. A 75-year-old woman presented with a spontaneous haematoma, high total protein level (157 g/L), low haemoglobin (86 g/L), and reduced platelets (103 x 10^9/L). SPE and immunofixation confirmed IgG lambda MM with a serum paraprotein concentration of 94 g/L. Plasmapheresis treatment reduced total protein levels to 87 g/L and serum paraprotein levels to 41 g/L. Bone marrow biopsy confirmed extensive clonal plasma cell infiltrate, consistent with plasma cell myeloma. These findings support the conclusion that MM can present with atypical symptoms, such as spontaneous haematomas, and that early recognition and treatment of HVS are crucial to prevent life-threatening complications.

This case underscores the need for clinicians to be vigilant for atypical presentations of MM. Awareness of symptoms such as unexplained haematomas, persistent anaemia, and elevated serum protein levels can prompt earlier diagnosis and intervention, potentially improving patient outcomes. Given the rarity and varied presentations of MM, it is imperative for clinicians to improve diagnostic accuracy and patient care.

## References

[1] Tathineni P, Cancarevic I, Malik BH (2020). Uncommon presentations of multiple myeloma. Cureus.

[2] Bladé J, Fernández de Larrea C, Rosiñol L, Cibeira MT, Jiménez R (2011). Soft-tissue plasmacytomas in multiple myeloma: incidence, mechanisms of extramedullary spread, and treatment approach. J Clin Oncol.

[3] Martins CP, Reigota R, Santos SD, Gonçalves DL, Ribeiro PM (2021). Hyperviscosity syndrome: a rare presentation of IgG multiple myeloma. Clin Case Rep Int.

[4] Rogers AP, Estes M (2023). Hyper-viscosity syndrome. StatPearls [Internet].

[5] Willrich MA, Katzmann JA (2016). Laboratory testing requirements for diagnosis and follow-up of multiple myeloma and related plasma cell dyscrasias. Clin Chem Lab Med.

[6] Gertz MA (2018). Acute hyperviscosity: syndromes and management. Blood.

[7] Molloy CB, Peck RA, Bonny SJ, Jowitt SN, Denton J, Freemont AJ, Ismail AA (2007). An unusual presentation of multiple myeloma: a case report. J Med Case Rep.

